# The Law as It Relates to the Physicians and the Buffalo Health Department

**Published:** 1912-04

**Authors:** Francis E. Fronczak

**Affiliations:** Health Commissioner; Buffalo


### BUFFALO MEDICAL JOURNAL

VOL. LXVII. APRIL, 1912. No. 9

### ORIGINAL COMMUNICATIONS

### The Law as it Relates to the Physicians and the Buffalo Health Department.

Dr. FRANCIS E. FRONCZAK, Health Commissioner,
Buffalo.

I HAVE been asked by various physicians on numerous occasions to prepare a paper on the duties of the physicians, as they relate to the Department of Health. Some have asked that I prepare a general paper on physicians and the law. I have devoted considerable time to the preparation of such a paper, and have made quite a study of the criminal, penal, and civil codes, the public health laws, the laws that relate to insanity, tuberculosis, the City Charter and Ordinances, and find that it will take a good sized book to cover all that relates to the physicians in the various statutes. Those who desire to make a more extensive study of the subject, I would refer to Taylor "On the Law and in its Relation to Physicians." To Purrington on "Legal Decisions, Medical." To Harold's "Manual on Legal Medicine." To Tidy on "Legal Medicine," and Allen McLane Hamilton, Peterson & Hains, and many others. The literature on this subject is not so meager as some might think.

In view of this, I will, of course, only dwell on matters which are binding in this City and State, and will start with the City Ordinances.

Every physician, accoucheur, midwife, undertaker, sexton or superintendent of any cemetery shall register his or her name in a book provided for this purpose in the Bureau of Vital Statistics, at the office of the Health Commissioner. Every physician before practicing in the City of Buffalo and County of Erie must register in the County Clerk's office.

Probably the question which is most often asked is what diseases are supposed to be infectious.

UNDER THE CITY ORDINANCES.

A. Contagious (very readily communicable): Measles, Rubella, (Rötheln) Scarlet Fever, Smallpox, Varicella (Chickenpox), Typhus Fever, Relapsing Fever, Bubonic Plague.

B. Communicable: Diphtheria (croup in all forms), Typhoid Fever, Asiatic Cholera, Anthrax, Glanders, Epidemic Cerebro-Spinal Meningitis, Leprosy, Tuberculosis (of any organ), Infectious Eye Disease (trachoma), Suppurative Conjunctivitis, Ophthalmia Neonatorum, Puerperal Septicaemia, Erysipelas, Whooping Cough, Poliomyelitis, Polioencephalitis.

C. Indirectly communicable (through intermediate host) Yellow Fever, Malarial Fever.

It is the duty of every physician upon discovering any case of infectious disease to report the same forthwith to the Health Commissioner. By forthwith is meant to telephone immediately upon discovery to the Health Department, which is open from 8:30 in the morning until 11 P. M.

The New York State Department requires the use of a one per cent. solution of Nitrate of Silver immediately after birth, as a prophylaxis against Ophthalmia Neonatorum. Complete outfits may be obtained gratis from the Buffalo Health Department and can be constantly carried in the obstetric bag.

Diphtheria—Quarantine will be maintained until two negative Cultures, the first by the attending physician and the second by an inspector from the Department, have been received.

Other children in the same family may be permitted to attend school, provided a culture from their throats show negative results, their clothes and bodies are disinfected, and they live away from the quarantined premises.

Scarlet Fever—The minimum period of quarantine is thirty-five (35) days, and as much longer as the case may require until recovery. Exposed children must be excluded from school for two weeks. The same period must be observed by those pupils who leave home for the purpose of attending school. After having been away from the quarantined premises for two weeks, a physician's certificate stating that he has examined the pupil and that he is free from disease, will be approved by the Department.

Whooping Cough—Minimum period of exclusion from school is eight weeks.

Measles, German Measles, Chicken Pox are excluded for a minimum of two weeks.

All others until recovery.

Typhoid Fever—Report all cases, regardless of Widal results.

Any officer or person in charge of any person, asylum, hospital or public or private institution of any kind shall report forthwith to the Health Commissioner any and every case of any infectious disease (except tuberculosis which must be reported within twenty-four hours), which shall arrive or be discovered, and shall, once a week, report all such cases, and upon death or recovery shall report the date and the details of the disposition of the case according to the rules and regulations of the Health Department.

The removal from one building to another of any person sick with any infectious disease is prohibited, except typhoid fever, malarial fever and tuberculosis, without permission from the Health Commissioner, and in the case of tuberculosis the premises from which the sick person was removed shall be forthwith disinfected before being again occupied and, within forty-eight (48) hours after such removal, notification (made compulsory by the State Law of the physician in attendance) shall be made to the Health Commissioner. Permission is sometimes granted to remove such person if a good and sufficient reason can be shown that it would be to the advantage of the community to remove such person from one house to another.

Every Smallpox case shall be removed to the quarantine hospital, unless such removal would place the life of the patient in jeopardy.

No person other than the attending physician shall enter or depart from a house in which is a person suffering from any of the diseases mentioned without permission from the Health Commissioner, except in cases of tuberculosis, typhoid fever, infectious eye diseases, puerperal septicaemia or malarial fever, and until such house has been disinfected according to the rules of the Health Department.

Removal of an infected person from one building to another, except to a hospital, is forbidden, without permission of the Health Commissioner.

Under the general title "Burial of the dead" we read that no death certificate shall be valid unless signed by the County Medical Examiner or a regularly licensed physician. By a decision of the Corporation Counsel this includes the Osteopaths.

A dead unembalmed body of a human being shall remain unburied no longer than three days, except by special permission from the Health Commissioner. Nor shall any dead body be left unburied for more than six days.

No body may be embalmed unless a physician issues a burial permit and also signs especially the permit providing that the body may be embalmed.

State Law prohibits the embalming of any dead body without a written certificate from the attending physician that there are no facts attending the illness and death of the person that would preclude such embalming from a medico-legal standpoint.

Physicians are often inquiring under what conditions it is possible to disinter or expose the remains of a dead person.

Ordinances provide that the remains of a person who shall have died of an infectious disease cannot be lawfully disinterred or exposed within ten years from burial, or any other human remains within one year after burial, without a permit from the

Commissioner of Health, and upon the payment of a fee of $5.00 for such permit. (This $5.00 of course must be turned into the City Treasury). Such disinterment or exposure shall be made only under the supervision of the Health Commissioner and pursuant to such rules and regulations as may be prescribed by the Department of Health.

File a certificate of every birth within three days, upon blanks furnished by the Bureau of Vital Statistics.

Premature births, or infants who die immediately after birth, require both a birth and a death certificate. A birth certificate must be filed of every infant that has breathed after birth.

Infants born dead must be reported on a still-birth certificate.

Death certificates must be made by the physician last in attendance upon the deceased, using blanks obtained from the Registrar of Vital Statistics. Every question should be answered, writing plainly and with unfading ink, as these are permanent records. No soiled or mutilated certificates will be received. A death certificate must be filed within twenty-four (24) hours, except in death from a contagious disease when it must be filed at once. The nomenclature adopted by the Bureau of Census, the New York State Department, and the Buffalo Health Department, must be followed.

Law prohibits the embalming of any dead body without a written certificate from the attending physician that there are no facts attending the illness and death of the person that would preclude such embalming from a medico-legal standpoint.

Under the City Charter, City Ordinances, and the State Law, it is a penal offense for any one to interfere with the Health Commissioner, or any one connected with the Health Department, when they are investigating any suspected violation of the health laws, and it is a penal offense (Penal Law Sec. 1741) to interfere with them in the performance of their duty, or to molest them in their work, and in the discharge of their duty, and one so doing is guilty of misdemeanor.

The Superintendent of a hospital, or the persons in charge of any hospital under the law, must report to the Health Commissioner any and all cases of infectious diseases that have been conveyed or carried to the hospital in any carriage, or other public conveyance, other than the ambulance, giving to the Commissioner the name of the driver, or person in charge of such vehicle, and must enter upon the books and records of such hospital the name of such driver or person in charge, or owner. Failure subjects the offender to a fine of not less than $25.00 or more than $100.00 for each and every offense.

Pupils residing in asylums or institutions shall on Monday of each week furnish a certificate from the head of such institution or attending physician to the effect that no contagious or infectious disease has existed in the same for the past week, and no pupil shall attend any school who resides in any public institution wherein a contagious or infectious disease has occurred, until he or she shall present a certificate from the institution, or the attending physician to the effect that no new case has occurred within ten days; that all cases have been removed to the hospital for contagious or infectious disease; that all approved methods of disinfection have been successfully employed and that no communication has been allowed between the attendants of isolated wards and those of the institution proper. Such certificate to be approved by the Health Commissioner. A violation of the above ordinance subjects the offender to a fine of from $50.00 to $250.00.

Under Section 58 of the Labor Law every physician attending or called in to visit a patient whom he believes to be suffering from poisoning from lead, phosphorus, arsenic or mercury, or their compounds, or from anthrax, or from compressed air illness, contracted as the result of the nature of the patient's employment, shall send to the Commissioner of Labor, at Albany, a notice stating the name and full postal address and place of employment of the patient and the disease from which, in the opinion of the medical practitioner, the patient is suffering, and failure to send such a notice is punishable by a fine not to exceed $10.00. This section took effect September 1st, 1911.

With the exception of the cities of New York, Brooklyn, Buffalo, Albany and Yonkers, physicians are entitled to receive twenty-five cents for reporting any contagious diseases, or filing of a birth or death certificate.

Under Section 313 of the Public Health Law all children admitted to institutions for orphans, destitute or vagrant children or juvenile delinquents must be examined by a physician, stating whether the child has diphtheria, scarlet fever, measles, whooping cough, or any contagious or infectious disease especially of the eyes and skin, which might be communicated to other inmates, specifying the physical and mental condition of the child, the presence of any indication of hereditary or other constitutional diseases, or any deformity or abnormal condition which may be found upon examination, and the physician must again examine the child after it is kept in strict quarantine the necessary number of days before the child can be admitted to come in contact with the other children.

Section 314 provides that such a physician shall, at least once a month thoroughly examine and inspect the entire institution, and report in writing to the board of managers or directors of the institution, the condition of the plumbing, sinks, water closets, urinals, privies and dormitories, the physical condition of the children, the existence of any contagious or infectious disease, particularly of the eye or skin, their food, clothing and cleanliness, and whether the officers have provided sufficient nurses, orderlies and attendants.

Section 315 makes it mandatory upon the physician of any institution in the state to notify in writing immediately the local board of health and the board of managers or the directors of any institution of any violation as regards the condition of the dormitory where beds must be separated by a passageway of not less than two feet in width, so that the air can circulate freely, and that there is 600 cubic feet of air space in the dormitory for either bed or occupant.

Sections 320 to 332 refer particularly to tuberculosis providing that all cases and all forms of tuberculosis, regardless of sputum examination or previous report of other physician, shall be reported to the Health Officer within twenty-four (24) hours after discovery. Laboratory results in any examination do not constitute a report of a case. In tuberculosis the following points are of special importance. The report must be made by physicians, also by persons in charge of hospitals, dispensaries, etc. The examination of sputum is made by the City Bacteriologist free of expense; all tuberculosis records are protected against public inspection; the courts having rules that they cannot even be used as evidence in a trial. In case of death or removal or vacation of any apartments or premises, the attending physician, or if no physician, the owner, lessee or occupant or other person in charge of such premises or apartments must notify the Health Department of such death, or removal within twenty-four (24) hours thereafter, and these apartments or premises so vacated cannot again be occupied until they are duly disinfected, cleansed or renovated. The Health Department should be notified when a patient discontinues treatment with a physician or when he moves to another part of the City, or out of the City. In reporting the cases the physician should state whether he is willing and able to attend to the enforcement of all precautionary measures.

Sputum cups, napkins and a complete outfit for the use of patients will be furnished by the Department of Health. Also circulars with complete information. In such cases where the attending physician is unwilling or unable to attend to the enforcement of precautionary measures, the tuberculosis inspectors will relieve him. Occupancy of any premises or apartments is prohibited until the order of the Health Commissioner is complied with.

Tuberculosis nurses will attend patients on request of the physician or when patients are in need of assistance.

Failure of a physician to perform duties, or making false reports, relative to tuberculosis, makes him guilty of a misdemeanor, and on conviction he may be subject to a fine of not more than $100.00.

Under Section 200 of the New York State Penal Code a physician or surgeon who being in the state of intoxication, without design to affect death, administers a poisonous drug or medicine or does anything else as such physician or surgeon and produces the death of another person is guilty of manslaughter in the second degree, and, if he injures his life, or his health is seriously affected, is guilty of misdemeanor.

It may surprise many a physician that under Section 191 of the Penal Code a person who provides, supplies, or administers to a woman, whether pregnant or not, or who prescribes for, or advises or procures a woman to take any medicine, drug, or substance, or who uses or employs or causes to be used or employed, any instrument or other means, with intent thereby to procure the miscarriage of a woman, unless the same is necessary to preserve her life, in case the death of the woman, or of any quick child of which she is pregnant, is thereby produced, is guilty of manslaughter in the first degree.

Under Section 294 a person is guilty of abortion even if he only prescribes, supplies or administers to a woman, or advises or causes any woman to take any medicine, drug, or substance, and is punishable by imprisonment in state prison for not more than four years or in a county for not more than one year. However, mere advice, but not followed by any action on the part of the woman based upon such advice does not make out the crime of abortion.

It is not essential to guilt that the woman should be quick with child, nor is it a defense that a harmless substance was administered provided the guilty intent existed. A physician was convicted in Iowa, though he pleaded that he gave the woman who was not quick with a child a substance which could do no harm to any woman, whether pregnant or not.

In another case it was declared by the judge that it is not necessary for the people to show that the use of the instrument was not necessary to preserve the life of the woman or of the child, but the burden of proving such necessity for its use rests upon the accused. Bradford v. People, 20 Hun. 309.

And under Section 297 of the Penal Code, any person who gives an instrument, medicine, or drug, or any other substance, with intent that the same may be unlawfully used in procuring the miscarriage of a woman is guilty of a felony.

Not only under City Ordinances, but under the Penal Code, a person who endeavors to conceal the birth of a child by any disposition of the dead body of the child, whether the child died before or after its birth is guilty of misdemeanor.

Under Section 318, any person who gives receipt, prescription, drug or medicine, which prevents conception, or causes unlawful abortion is guilty of misdemeanor and may be fined not more than $1,000, or sent to prison for one year.

It is even forbidden to exhibit or discuss offensive objects, such as unnatural and monstrous births, even though the pictures are not immoral. It is enough to expose to public view in a public place, so that it might probably be seen by the public, and does not depend upon the number of persons to whom the exposure is made.

It may surprise many, though in this country it seldom is applied, that a physician who acts as a second or a surgeon advises or abets or is present at a duel or combat that has been previously agreed to is punishable by an imprisonment for not more than seven years.

Very often the Department of Health is asked whether or not a physician has the right to dissect the dead body of a human being. This right exists only in cases prescribed by special statutes, or where a coroner or medical examiner is authorized by law, or where the husband, wife, or next of kin of the deceased being charged by the law with the duty of burial may authorize dissection for the purpose of ascertaining the cause of death and no further. I would advise you in these cases always to get your authorities in writing and hang on to it, because it may come handy within the statutes of limitation. Of course the District Attorney has authority to order dissection whenever there is suspicion of crime being committed.

Any person who makes, causes or procures any dissection, except by authority of the law, or in pursuance of a permission given by the deceased is guilty of misdemeanor. And the following section provides that the remains after dissection must be buried.

Of course all physicians know that body stealing is prohibited. It is not practiced now, and did not occur, as much as was popularly surmised, formerly. The law provides for imprisonment for not more than five years or a fine not to exceed $1,000, or both for this offense.

A person who willfully opposes or obstructs a health officer or official charged with the enforcement of the health laws in performing any legal duty is guilty of misdemeanor. (Section 396 Penal Code).

Section 397 provides that any person who wilfully violates or refuses or omits to comply with any lawful order or regulation prescribed by any local board of health or local health officer is guilty of a misdemeanor, and that any person who wilfully violates any provision of the health laws, or any regulations so established is liable to imprisonment not to exceed one year, or by a fine not to exceed $2,000 or both. Under this section any physician who does not report contagious and infectious diseases, as provided by the rules and regulations of the Health Department, or does not report births, within the time as prescribed by the law may be indicted by the grand jury.

Quite often we are asked whether or not a physician may maintain a private insane asylum, or an institution for the care or treatment of unsound minds. The Penal Code says that a license must be granted, and the law relating to insanity, No. 59, says that the Commissioner of Lunacy must first examine the place and may grant a license providing it fully complies with the rules and regulations of that commission. Any one who omits to have such examination made of such an institution without a license is guilty of a misdemeanor.

Under the Code of Criminal Procedure if a female, convicted and sentenced to punishment by death, is pregnant, she must be examined by a jury of six physicians to inquire into her pregnancy, and the physician so acting as a juror need not be qualified to serve as a juror in a court of record.

Whenever the death penalty is carried out upon a criminal in this State there must be two physicians present at the execution, and immediately after the execution a post-mortem examination of the body of the convict must be made by the physicians present, and their report must be in writing, stating the nature of the examination, and that the person was executed according to the law.

Another matter which may interest a physician is that there is a provision under the Code forbidding the exhibiting of a person affected with a contagious disease in a public place, unless such exposure is necessary to remove him, and this removal must be done in a manner not dangerous to public health. Any one who wilfully exposes himself to another is guilty of a misdemeanor.

